# Knowledge, Attitudes, Practices and Health Beliefs toward Leptospirosis among Urban and Rural Communities in Northeastern Malaysia

**DOI:** 10.3390/ijerph15112425

**Published:** 2018-11-01

**Authors:** Pathman A, Aziah BD, Zahiruddin WM, Mohd Nazri S, Sukeri S, Tengku Zetty TJ, Hamat RA, Malina O, Norazlin I, Zawaha I, Zainudin AW

**Affiliations:** 1 Department of Community Medicine, School of Medical Sciences, Universiti Sains Malaysia, Kubang Kerian 16150, Kelantan, Malaysia; pathman_19@hotmail.com (P.A.); drzahir@usm.my (Z.W.M.); drnazri@usm.my (M.N.S.); surianti@usm.my (S.S.); norazlin@usm.my (N.I.); 2 Department of Medical Microbiology and Parasitology, Faculty of Medicine and Health Sciences, Universiti Putra Malaysia, UPM Serdang, Selangor 43400, Malaysia; tengkuzetty@upm.edu.my (T.Z.T.J.); rukman@upm.edu.my (H.R.A.); malinaosman@upm.edu.my (M.O.); 3 Health Promotion Unit, Penang State Health Department, Floor 7, Bangunan Persekutuan, Jalan Anson, 10400 Penang, Malaysia; hjhzawaha@yahoo.com; 4 Health Department of Federal Territory Kuala Lumpur and Putrajaya, Jalan Cenderasari, Kuala Lumpur 50590, Malaysia; zwah58@gmail.com

**Keywords:** knowledge, attitude, belief, practice, leptospirosis, urban, rural

## Abstract

Background: Leptospirosis is a zoonotic disease with a worldwide distribution, especially in developing countries such as Malaysia. This study was designed to explore the knowledge, attitudes, beliefs and practices (KABP) toward leptospirosis among the communities in northeastern Malaysia and to determine the sociodemographic factors associated with the KABP toward leptospirosis. A cross-sectional study using a stratified sampling method was conducted among 214 individuals in four locales in northeastern Malaysia. Methods: A cross-sectional study was conducted among 214 respondents in northeastern Malaysia using a multi-stage stratified random sampling method. The study population was divided into two groups based on geographical locations: urban and rural. All data were entered and analyzed using the IBM Statistics for Social Sciences (SPSS) version 22.0 software for Windows (IBM, Armonk, NY, USA). The continuous variables were presented using mean and standard deviation (SD), whereas the categorical variables were described using frequency and percentage. Multiple logistic regression was performed to determine the associated factors for good KABP toward leptospirosis among the respondents. Results: It was found that 52.8% of respondents had good knowledge, 84.6% had positive attitudes, 59.8% had positive beliefs, and 53.7% had satisfactory practices. There were no significant sociodemographic factors associated with knowledge and practice, except for educational status, which was significant in the attitude and belief domains. Those with higher education exhibited better attitudes (Odds Ratio (OR) 3.329; 95% Coefficient Interval (CI): 1.140, 9.723; *p* = 0.028) and beliefs (OR 3.748; 95% CI: 1.485, 9.459; *p* = 0.005). The communities in northeastern Malaysia generally have good knowledge and a high level of positive attitude; however, this attitude cannot be transformed into practice as the number of people with satisfactory practice habits is much lower compared to those with positive attitudes. As for the belief domain, the communities must have positive beliefs to perceive the threat of the disease. Conclusions: Our current health program on preventing leptospirosis is good in creating awareness and a positive attitude among the communities, but is not sufficient in promoting satisfactory practice habits. In conclusion, more attention needs to be paid to promoting satisfactory practice habits among the communities, as they already possess good knowledge and positive attitudes and beliefs.

## 1. Background

Leptospirosis is a zoonotic disease with a worldwide distribution, especially in developing countries [1]. Annually, large outbreaks occur in tropical and subtropical climates which promote favorable conditions for transmission of the disease. Leptospirosis is a bacterial infection caused by a species of pathogenic Leptospira genus called spirochetes [2]. These spirochetes are known as *Leptospira interrogans* [3]. This contagious bacterium is transmitted from infected animals to humans [2]. The socioeconomic status, economy, occupation, recreational activities, association with animals, climate, and rainfall are significantly interrelated with the occurrence of leptospirosis infection [4].

In recent years, the number of leptospirosis cases has been increasing significantly in Malaysia [5]. Preventive measures have been taken, but they have not solved the problem. In order to understand leptospirosis and suggest possible strategies to control and prevent the disease, individuals need to have basic knowledge about it. Knowledge, attitude and practice (KAP) are highly associated with the incidence of many infectious diseases, but KAP is still considered low even in the high-risk groups [6]. Effective intervention requires basic information related to sociodemographic components, risk perception (belief), and preventive practice against rodent-borne infections, particularly leptospirosis; this information remains very limited in northeastern Malaysia.

Leptospirosis occurs throughout the year in Malaysia. Contrary to common belief, leptospirosis is not a new disease; the first known case of human leptospirosis was reported by Fletcher in 1927, indicating leptospirosis as an endemic disease in Malaysia. Due to the significant increase in the number of cases, leptospirosis became a notifiable disease in 2011 [7]. Distribution of leptospirosis in Malaysia for the year 2015 shows the highest incidence (21.4%) in northeastern Malaysia in comparison to the other states. The number of leptospirosis cases and deaths from January 2015 to July 2015 were 5370 and 30, respectively [7].

Several KAP studies have been conducted prior to the current one, most in high-risk groups, but no study has been done among the communities in northeastern Malaysia. Furthermore, this study incorporated the belief aspect, which is considered important according to the latest findings by the Ministry of Health Malaysia. It is also one of the main elements in the Health Belief Model (HBM). The determination of knowledge, attitude, belief and practice (KABP), and its associated factors, is crucial to the prevention of leptospirosis. As the management of this disease is challenging, and there is no proven vaccine available, a behavioral approach with proper understanding of social background and health beliefs has become a major priority in reducing mortality and morbidity due to leptospirosis [8].

## 2. Methods

### 2.1. Study Design and Selection of Participants

A cross-sectional study was conducted among 214 respondents in northeastern Malaysia using a multistage stratified random sampling method. Sample size was calculated using a single proportion formula with the proportion of good knowledge as 52% [9]. The study population was divided into two groups based on geographical locations: urban and rural. Two localities were chosen for each urban and rural area. Simple random sampling was performed, and 55 households from each locality were selected.

### 2.2. Research Tool

A self-administered questionnaire was developed based on a literature review of relevant studies and previous questionnaires. A panel of experts, including an epidemiologist, microbiologist, statistician, infectious disease physician, health educationist, and other relevant experts, was involved in developing the questionnaire in the local Malay language. A pilot study was conducted among 150 respondents to examine the test–retest reliability and internal consistency of the questionnaire. Internal consistency was measured by intraclass correlation coefficient (ICC) and kappa analysis of the categorical items. The exploratory factor analysis (EFA) was then calculated. For the knowledge domain, which had eight subdomains, the difficulty discrimination index ranged from 0.37 to 23.64, and Cronbach’s alpha was 0.86 (95% CI: 0.83, 0.90). For the attitude domain, which had eight subdomains, the factor loading ranged from 0.12 to 0.88, and Cronbach’s alpha was 0.76 (95% CI: 0.68, 0.83). For the practice domain, there were 10 subdomains with the factor loading ranging from 0.12 to 1.74, and Cronbach’s alpha was 0.81 (95% CI: 0.71, 0.90). 

The newly developed and validated KABP Questionnaire was used as a tool in this study. The questions were divided into two main sections, A and B. For Section A, information on the personal, occupational and environmental factors of the respondents was obtained. Section B covered the KABP of the respondents. The cut-off point of 80% or more was regarded as having a good knowledge, positive attitude, positive belief, and satisfactory practice, based on the decision agreed upon by the researchers and expert opinion.

### 2.3. Data Analysis

All data were entered and analyzed using the IBM Statistics for Social Sciences (SPSS) version 22.0 software for Windows (IBM, Armonk, NY, USA). Data were checked and cleaned. The continuous variables were presented using mean and standard deviation (SD), whereas the categorical variables were described using frequency and percentage. Multiple logistic regression was performed to determine the associated factors for good KABP toward leptospirosis among the respondents. A *p*-value of less than 0.05 was judged to be statistically significant.

## 3. Results

### Sociodemographic Characteristics

A total of 214 respondents participated in the present study yielding a response rate of 97.3% (the required sample with a 10% nonresponse rate was 218). All respondents were Malays, and females (51.9%) outnumbered the males (48.1%). The mean (SD) age of the respondents was 43.5 (±15.7) years of age. Table 1 shows the sociodemographic characteristics of the respondents in the urban and rural areas.

The proportion of good knowledge, positive attitude, positive belief and satisfactory practice is shown in Table 2.

Table 3, Table 4, Table 5 and Table 6 show the significant associated factors for good knowledge, positive attitude, positive belief and satisfactory practice toward leptospirosis, respectively.

## 4. Discussion

A total of 52.8% of the respondents had a good knowledge score. This is probably due to government initiatives to prevent the disease by advertising about it in the mass media. Furthermore, healthcare workers are very active and deliver informational health talks and disseminate information on leptospirosis in northeastern Malaysia where the disease is widespread. A similar finding was noted in Jamaica where 97% of respondents in a particular community had heard of the disease because of its prevalence in their area [10]. A study done in Trinidad and Tobago, however, found that only about 25% of the respondents had ever heard of the disease [9]. Additionally, a different study done among town service workers in northeastern Malaysia showed a low percentage (13%) of those who had heard of leptospirosis [11]. Perhaps, the public health approach toward the communities is more effective compared to the focus target groups, and that is the reason the communities seem to a have better knowledge of the disease when compared to other target groups. 

Knowledge for the “prevention methods” subdomain showed that the majority of the respondents knew how to prevent leptospirosis infection mainly by avoiding contact with rats and keeping a safe home and workplace, avoiding wading in flood water, drinking and eating safe food and water, and wearing proper protective appliances. Knowledge of using proper personal protective equipment (PPE) plays an important role in preventing the disease [12,13]. This shows that the communities have good knowledge of preventive measures, similar to the communities in a study done in Malaysia among oil palm plantation workers where more than 60% had knowledge of PPE [14]. From this study, it was evident that most respondents identified leptospirosis from its local name, which is *penyakit kencing tikus*. 

The attitude domain had the highest score among the others in which 84.6% of the respondents had a positive attitude. The majority of the respondents strongly agreed that they will wear PPE while handling rubbish. This attitude might not be directly related to leptospirosis but more toward a preventive attitude about getting any form of disease, as it is a widely known fact that improper rubbish handling spreads bacteria and one can be ill if the bacteria manage to spread to humans. 

Most of the respondents were bothered with the presence of rats around their housing area. This shows adequate knowledge was present among the respondents and promoted a positive attitude toward rat control. A similar finding was noted where oil palm plantation workers said that the presence of accumulated garbage and the presence of rats and other rodents increase the risk of transmission of leptospirosis [14]. The majority of the respondents also agreed that they have to see the doctor if they fall sick and that they must follow the doctor’s instructions until they fully recover. 

The positive attitude among the communities in the study area might be due to the fact that sufficient knowledge on the routes of transmission of the disease encouraged the communities to have a positive preventive attitude. Knowing the symptoms of leptospirosis also made it easier for respondents to know when they have to see a doctor because they are aware of the severity of the disease. It also shows that the health sector is doing a good job in reaching out to the communities to spread the information on diseases such as leptospirosis.

Only 53.7% of respondents had good practice. This result is similar to a study done in a highly endemic area in Sri Lanka where most of the respondents had high knowledge but poor practice [15]. Many respondents agreed that they should wear gloves while handling garbage, but, when it comes to practice, not many respondents do this. Only half of the respondents reported that they never threw out rubbish if there was a wound on their legs or hands and that they washed their hands each time after throwing out rubbish. This finding can be related to the “complication” subdomain in the knowledge section, where many of the respondents, though aware of the disease, did not know about the complications of the disease. This gap in knowledge is leading them to have bad practices, as they are not equipped with the knowledge of the actual severity of the disease.

This is where the belief aspect of the community comes into place. When the communities do not have sufficient belief toward the disease, that is when they will take things for granted and not have good practice for disease prevention. These findings were similar to a study done in Sri Lanka, where, despite having good knowledge, adolescents did not have good practice because of knowledge gaps in certain areas [15]. 

Health belief is a new aspect which is now being studied in the area of leptospirosis. In the present study, about 60% of respondents had good belief. Almost half of the respondents had the belief that leptospirosis can be treated traditionally. According to the National Health and Morbidity Survey (2015), many Malaysians choose traditional medicine as their first line of treatment before seeking modern medical services. In contrast to sufficient knowledge among the respondents, about 40% had the belief that leptospirosis was not dangerous and that they were not at risk of contracting the disease. This might be the result of having insufficient knowledge of the severity and the complications of the disease. Healthy individuals might think that they are not at risk of transmitting the disease and that only people who are immunocompromised or who are elderly are at risk of developing the disease. 

More effort should be exerted by the relevant sectors to change the pre-existing beliefs that the communities have. Most of the beliefs that are present in the current generation are due to cultural aspects and the influence of parents and friends, and these need to be changed in order to have better outcomes and to reduce the prevalence of leptospirosis in the future. Previous studies also identified the importance of health promotions as contributing factors to increase the community’s perceived threat of the disease by the means of video-based education or other real-life scenarios [16].

The only variable that was significant for positive attitude and positive belief was education level. Although the knowledge of the respondents is not significantly related to the education level, we can see that the attitude and belief of an individual need to be cultivated from when that person is young, and this is where education level plays a big role. When they have higher education levels, this means that individuals have been attending school for a longer time and that the proper attitudes and beliefs about certain diseases, especially infectious diseases, have been instilled in them. People with lower education levels might have sufficient knowledge, but this does not mean that they have the proper understanding to translate it into proper attitudes and beliefs. In addition, cultural norms also play an important role in the attitudes and beliefs of an individual. Similar data were found among butchers in Jamaica, where the majority of them attended only primary school and had poor attitudes toward infectious diseases [17]. Another study recently done in Kuantan, Malaysia suggests that people with lower education levels have lower attitude outcomes as well [18].

Education level showed no significant association with the satisfactory practice toward leptospirosis among the respondents. Perhaps, those with higher educational levels did not have time to practice good habits which would prevent the disease. The highly educated population spends more time at work, which means they have less time to clean their houses and also to throw out rubbish on a frequent basis. Another aspect is that people with high levels of education might think that they know a lot and that they do not need to see a doctor if they become sick. Moreover, having a good education does not mean that an individual has a good income. Some practice habits require a certain amount of money to be spent on personal protective equipment, such as goggles and boots, which means, although having sufficient attitudes and beliefs, individuals cannot translate these into practice due to financial constraints. In contrast, a study in Thailand showed that educational status did play a significant role in the outcome of practice [19]. The reason for this might be that the respondents did not have sufficient knowledge to put into practice, despite having sufficient financial support.

## 5. Conclusions

This study demonstrated an interesting pattern in the KAPB toward leptospirosis among the communities in northeastern Malaysia. Exploring the KABP and its associated factors will help policymakers and healthcare workers to better understand the community and its actual needs in order to promote better practice habits to reduce the incidence of leptospirosis in the future. From this study, it is evident that a highly positive attitude score alone is not sufficient to translate into behavioral practice. Community-based health education and promotion activities are needed to increase knowledge about leptospirosis and, subsequently, improve preventive practices. The collaboration among the health sector and other sectors, such as local governments, private enterprises, policymakers and nongovernment organizations, should be encouraged to strengthen the overall health knowledge, attitudes, practices and beliefs of the community toward leptospirosis. 

## Figures and Tables

**Table 1:** Sociodemographic characteristics of the respondents (*N* = 214).

Variables	Rural (*n* = 107)		Urban (*n* = 107)	
	Frequency (%)	Mean (SD)	Frequency (%)	Mean (SD)
Age (years)		38.04 (14.80)		48.87 (14.80)
Gender				
Male	36 (35.0)		67 (65.0)	
Female	71 (64.0)		40 (36.0)	
Marital Status				
Married	77 (46.7)		88 (82.2)	
Unmarried	19 (57.6)		14 (13.1)	
Divorcee	11 (69.0)		5 (4.7)	
No. of Children		3.05 (2.64)		3.97 (2.59)
Monthly Income		457.94 (539.49)		1152.68 (1464.53)
Education Level				
Primary	17 (47.2)		19 (52.8)	
Lower Secondary	22 (59.5)		15 (40.5)	
Upper Secondary	52 (57.1)		39 (42.9)	
Form Six	13 (34.2)		25 (65.8)	
Tertiary	3 (25.0)		9 (75.0)	

**Table 2:** Proportion of good knowledge, positive attitude, positive belief and satisfactory practice (*N* = 214).

Variable	Percentage (%)
Good Knowledge	52.8
Positive Attitude	84.6
Positive Belief	59.8
Satisfactory Practice	53.7

**Table 3:** Associated factors for good knowledge toward leptospirosis using multiple logistic regression (*N* = 214).

Variable	Crude OR (95% CI) ^a^	Adjusted OR (95% CI) ^b^	Wald Statistic *(df)*	*p*-Value
Marital Status Unmarried Married	1 1.899 (0.891, 4.050)	1 2.037 (0.943, 4.401)	3.278	0.07
Education Status Primary Secondary Tertiary	1 1.417 (0.674, 2.979) 1.726 (0.727, 4.098)	1 1.445 (0.684, 3.053) 1.910 (0.792, 4.603)	0.929 2.079	0.34 0.15

^a^ Simple logistic regression; ^b^ multiple logistic regression. Hosmer-Lemeshow test (*p*-value = 0.992). Classification table (overall correctly classified percentage = 57.6%). Interaction terms have been checked and not found. Area under ROC (Receiver Operating Characteristic) curve (57.9%, *p*-value = 0.047).

**Table 4:** Associated factors for positive attitude toward leptospirosis using multiple logistic regression (*N* = 214).

Variable	Crude OR (95% CI) ^a^	Adjusted OR (95% CI) ^b^	Wald Stat (*df*)	*p*-Value
Geography Rural Urban	1 0.602 (0.282, 1.282)	1 0.620 (0.281, 1.367)	1.404	0.23
Education Status Primary Secondary Tertiary	1 4.071 (1.676, 9.893) 3.071 (1.067, 8.843)	1 3.919 (1.604, 9.573) 3.329 (1.140, 9.723)	8.981 4.837	0.03 0.02

^a^ Simple logistic regression; ^b^ multiple logistic regression. Hosmer-Lemeshow test (*p*-value = 0.645). Classification table (overall correctly classified percentage = 84.6%). Interaction terms have been checked and not found. Area under ROC curve (66.1%, *p*-value 0.03).

**Table 5:** Associated factors for positive belief toward leptospirosis using multiple logistic regression (*N* = 214).

Variable	Crude OR (95% CI)	Adjusted OR (95% CI)	Wald Stat (*df*)	*p*-Value
Gender Male Female	1 0.709 (0.409, 1.229)	1 0.804 (0.442, 1.462)	0.511	0.48
Geography Rural Urban	1 1.598 (0.921, 2.772)	1 0.804 (0.442, 1.462)	2.093	0.15
Marital Status Unmarried Married	1 0.601 (0.270, 1.335)	1 0.630 (0.273, 1.454)	1.172	0.28
Education Status Primary Secondary Tertiary	1 2.949 (1.367, 6.359) 4.128 (1.661, 10.257)	1 3.162 (1.445, 6.917) 3.748 (1.485, 9.459)	8.307 7.822	<0.01 <0.01

^a^ Simple logistic regression; ^b^ multiple logistic regression. Hosmer-Lemeshow test (*p*-value = 0.525). Classification table (overall correctly classified percentage = 64.2%). Interaction terms have been checked and not found. Area under ROC curve (65.2%, *p*-value 0.01).

**Table 6:** Associated factors for satisfactory practice toward leptospirosis using multiple logistic regression (*N* = 214).

Variable	Crude OR (95% CI) ^a^	Adjusted OR (95% CI) ^b^	Wald Stat (*df*)	*p*-Value
Geography Rural Urban	1 1.514 (0.882, 2.600)	1 1.371 (0.787, 2.388)	1.239	0.27
Employment status Unemployed Employed	1 1.736 (1.009, 2.988)	1 1.627 (0.934, 2.835)	2.950	0.09

^a^ Simple logistic regression; ^b^ multiple logistic regression. Hosmer-Lemeshow test (*p*-value = 0.859). Classification table (overall correctly classified percentage = 59.2%). Interaction terms have been checked and not found. Area under ROC curve (59.1%, *p*-value = 0.029).

## Declarations:

Ethics approval and consent to participate: Ethics approval was obtained from Research and Ethics Committee of Universiti Sains Malaysia (USM/JEPeM/15120522). All participants were briefed prior to the distribution of questionnaire and a written consent was obtained from all participants.

## Availability of Data and Material

All data and material is with the principal investigator and main author. All softcopy of data and material are available if needed and hardcopy of questionnaire and consent are with the principal investigator.

## Abbreviations

AOR	adjusted odds ratio
FGD	focus group discussion
HIV	human immunodeficiency virus
KABP	knowledge, attitudes, beliefs and practices
KAP	knowledge, attitudes and practices
KKM	Kementerian Kesihatan Malaysia
MOH	Ministry of Health
OR	odds ratio
PPE	personal protective equipment
WHO	World Health Organisation

## References

[1] Pappas G., Papadimitriou P., Siozopoulou V., Christou L., Akritidis N. (2008). The globalization of leptospirosis: Worldwide incidence trends. Int. J. Infect. Dis..

[2] Haake D.A., Levett P.N., Adler B. (2015). Leptospirosis in Humans. Leptospira and Leptospirosis. Current Topics in Microbiology and Immunology.

[3] Adler B., de la Peña Moctezuma A. (2010). Leptospira and leptospirosis. Vet. Microbiol..

[4] Lau C., Smythe L., Weinstein P. (2010). Leptospirosis: An emerging disease in travellers. Travel Med. Infect. Dis..

[5] Benacer D., Thong K.L., Min N.C., Verasahib K.B., Galloway R.L., Hartskeerl R.A., Zain S.N.M. (2016). Epidemiology of human leptospirosis in Malaysia, 2004–2012. Acta Trop..

[6] Victoriano A., Smythe L.D., Gloriani-Barzaga N., Cavinta L.L., Kasai T., Limpakarnjanarat K., Coulombe C. (2009). Leptospirosis in the Asia Pacific region. BMC Infect. Dis..

[7] Said Z.M. (2015). Infectious Disease Data 2015.

[8] Erasmus V., Brouwer W., Van Beeck E., Oenema A., Daha T., Richardus J., Brug J. (2009). A Qualitative Exploration of Reasons for Poor Hand Hygiene Among Hospital Workers Lack of Positive Role Models and of Convincing Evidence That Hand Hygiene Prevents Cross-Infection. Infect. Control Hosp. Epidemiol..

[9] Mohan A.R., Chadee D.D. (2011). Knowledge, attitudes and practices of Trinidadian households regarding leptospirosis and related matters. Int. Health.

[10] Allwood P., Muñoz-Zanzi C., Chang M., Brown P.D. (2014). Knowledge, perceptions, and environmental risk factors among Jamaican households with a history of leptospirosis. J. Infect. Public Health.

[11] Sulong M.R., Shafei M.N., Yaacob N.A., Hassan H., Daud A., Mohamad W.M.Z.W., Abdullah M.R. (2011). Risk Factors Associated with Leptospirosis among Town Service Workers. Int. Med. J..

[12] Dias J.P., Teixeira M.G., Costa M.C.N., Mendes C.M.C., Guimarães P., Reis M.G., Barreto M.L. (2007). Factors associated with Leptospira sp infection in a large urban center in northeastern Brazil. Rev. Soci. Bras. Med. Trop..

[13] Gaynor K., Katz A.R., Park S.Y., Nakata M., Clark T.A., Effler P.V. (2007). Leptospirosis on Oahu: An outbreak associated with flooding of a university campus. Am. J. Trop. Med. Hyg..

[14] Ridzuan J., Aziah B.D., Zahiruddin W.M. (2016). The Occupational Hazard Study for Leptospirosis among Agriculture Workers. Int. J. Collab. Res. Intern. Med. Public Health.

[15] Samarakoon Y., Gunawardena N. (2013). Knowledge and self-reported practices regarding leptospirosis among adolescent school children in a highly endemic rural area in Sri Lanka. R. Remote Health.

[16] Swai E.S., Schoonman L., Daborn C. (2010). Knowledge and attitude towards zoonoses among animal health workers and livestock keepers in Arusha and Tanga, Tanzania. Tanzan. J. Health Res..

[17] Brown P., McKenzie M., Pinnock M., McGrowder D. (2011). Environmental risk factors associated with leptospirosis among butchers and their associates in Jamaica. Int. J. Occup. Environ. Med..

[18] Edre M., Hayati K., Salmiah M.S., Sharifah Norkhadijah S.I. (2015). A Case Control Study on Factors Associated with Leptospirosis Infection Among Residents In Flood-Prone Area, Kuantan: A Geographical Information System-Based Approach. Int. J. Public Health Clin. Sci..

[19] Wiwanitkit V. (2006). A note from a survey of some knowledge aspects of leptospirosis among a sample of rural villagers in the highly endemic area, Thailand. R. Remote Health.

